# Rainfall variability patterns in Nigeria during the rainy season

**DOI:** 10.1038/s41598-023-34970-7

**Published:** 2023-05-16

**Authors:** Chibuike Chiedozie Ibebuchi, Itohan-Osa Abu

**Affiliations:** 1 Department of Geography, Kent State University, Kent, OH USA; 2 Department of Remote Sensing, Institute for Geography and Geology, University of Würzburg, Am Hubland, 97074 Würzburg, Germany; 3 ClimRISE Lab, Kent State University, Kent, OH USA

**Keywords:** Atmospheric science, Atmospheric dynamics

## Abstract

To enhance the physical understanding of the circulation patterns associated with rainfall variations in Nigeria, we spatially decomposed rainfall during the rainy season and uncovered the asymmetric atmospheric circulation patterns driving wet and dry regimes in specific parts of Nigeria. Also, we examined linear trends in rainfall and the circulation patterns driving the trends. Our result shows that during the analysis period (1979–2022), northern part of Nigeria has coherent rainfall anomaly that is coupled with rainfall variations over the Sahel (Pearson correlation coefficient (r) is 0.55), and sea surface temperature anomalies (SSTa) in the global oceans (r = $\left|0.5\right|$). The negative phases of the Pacific Decadal Oscillation, North Atlantic Oscillation, and the North Pacific Oscillation; and the positive phases of the Atlantic Multidecadal Oscillation and the Pacific warm pool are associated with rainfall increase over the northern part of Nigeria. Owing to the increasing trend in SSTa over the Mediterranean and the adjacent oceans, implying the weakening of dry northerly winds penetrating northern Nigeria, the rainfall trend is significantly positive in the northern part of Nigeria during the rainy season—with an increase of about 2–4 mm/year, especially during August. The circulation patterns associated with rainfall formation at the western and southeastern parts of Nigeria are shown to be associated with SSTa over the tropical Atlantic Ocean, south coast of Nigeria (r = $\left|0.4\right|$). Moreover, rainfall shows a negative trend, with a decrease of about 5 mm/year, in the southeastern parts of Nigeria, which can be linked to the warming trend over the Gulf of Guinea.

## Introduction

Climate change and natural climate variability are the major causes of weather extremes such as heavy rainfall and drought conditions^1,2^. Regional climate variability is driven by anomalies in the large-scale ocean and atmospheric circulations that modify regional atmospheric transport^3,4^. The climatic drivers are modes of climate variability associated with defined pattern configuration and exist over defined regions; also, they are mostly asymmetric in their morphology and physical characteristics, therefore inducing opposing weather conditions^5,6^. For example, the El Niño Southern Oscillation mode exists over the tropical Pacific Ocean with an asymmetry of two opposing phases—El Niño and La Niña that induces opposing weather conditions. The growth and anomalies in the climatic modes exist mostly due to internal ocean–atmosphere dynamics^7^. With an increase in weather extremes around the globe, researches aim at detangling weather extremes caused by anomalies in climate drivers from weather extremes due to anthropogenic climate change (i.e., emission of greenhouse gases), as well as how anthropogenic climate change influences the frequency, amplitude and pattern configuration of the climate drivers. In that paradigm, while anomalies in climate drivers are due to natural phenomena, the greenhouse gas effect can still modify the nature of climate drivers, thereby exacerbating climate extremes^8–10^. Therefore, a major goal of climate science in curtailing climate extremes and their societal disasters is by ascertaining the climate drivers that influence climate variability over a given region, enhance predictions of regional weather extremes due to anomalies in the climate drivers and investigate the contributions of anthropogenic climate change in the regional weather extremes. Given the recent hydrological extremes in Nigeria^11^, this study focuses on improving the understanding of the large-scale circulation patterns driving rainfall changes over Nigeria.

**Figure 1:**
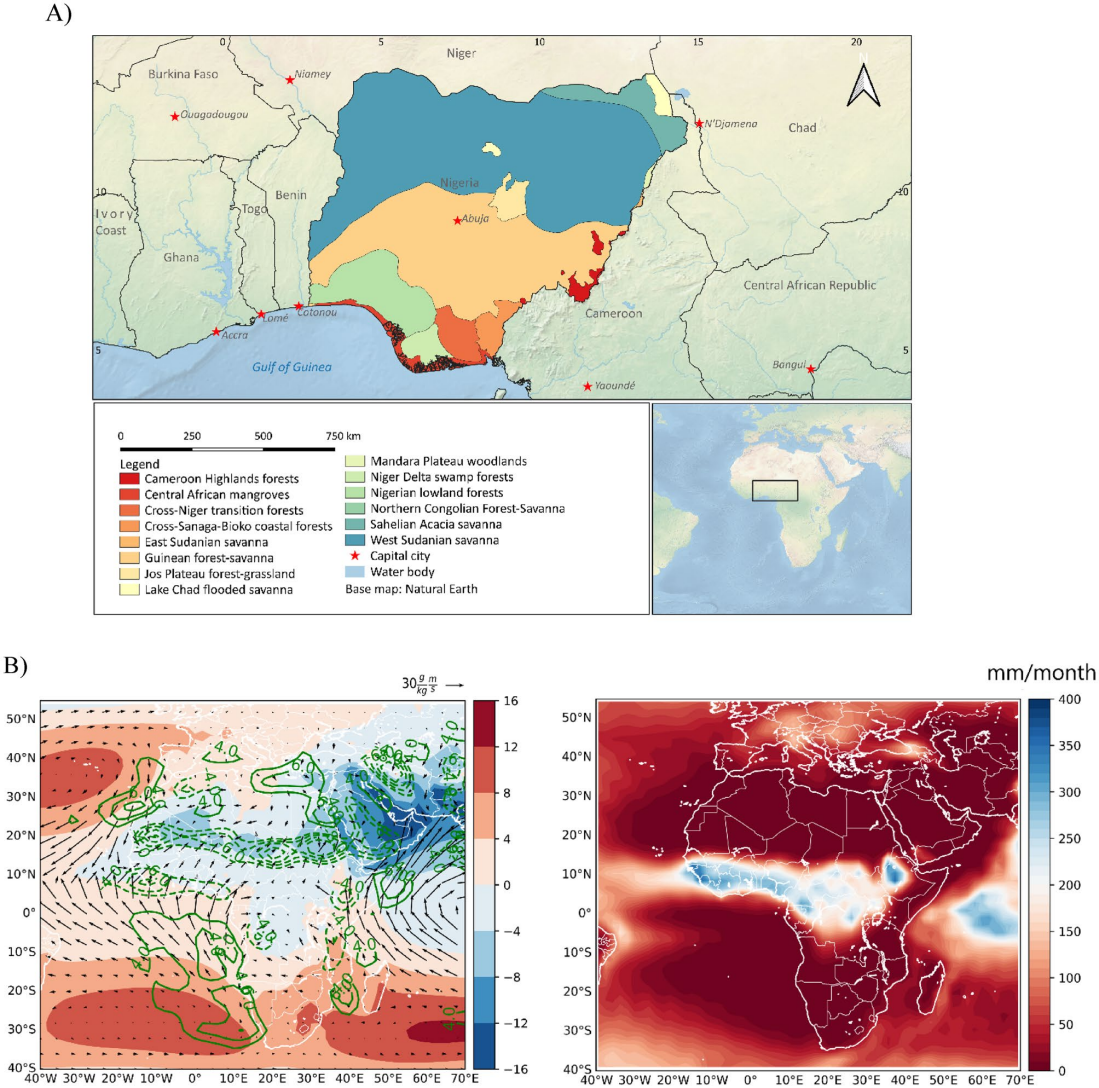
Classification of the ecoregions in Nigeria according to^16^ (**A**), and the climatology of standardized sea level pressure, 850 hPa vertical velocity, 10 m moisture flux (**B**, left panel), and rainfall (**B**, right panel) during July to September. The Black Contour line is vertical velocity, in m/s, color is SLP, and the green vector is 10 m moisture fluxes. Thick contours (positive values) designate downward motion. Figures 1A was created using QGIS version 3.30 available at https://www.qgis.org/en/site/forusers/download.html; and 1B was created using python 3.8.8 available at https://www.python.org/downloads/release/python-388/.

Nigeria is a country in West Africa that is characterized by a wide variety of ecoregions (Fig. 1A). There are two major rainfall seasons in Nigeria—the dry season (typically from November to March) and the rainy season (typically from April to October). The north and south seasonal migration of the Inter-tropical Convergence Zone (ITCZ) following changes in the region of maximum diabatic heating^12^, in addition to sea surface temperature anomalies (SSTa) at the adjacent oceans are among the principal factors that control the seasonal rainfall variability in Nigeria^13^. Latitudinal changes in the ITCZ are accompanied by variations in the convergence of northeasterly winds advecting through the Sahara and southwesterly winds defining the West Africa Monsoon system, which plays a vital role in mean rainfall variations over West Africa^12–14^. Figure 1B shows the climatology of sea level pressure (SLP), 850 hPa vertical velocity (negative values designate upward motion), 10 m moisture flux and rainfall, for the 1981–2010 period, from the ERA5 reanalysis. From Fig. 1B, during the peak of the rainy season in Nigeria (July–September), the region of maximum convergence (ITCZ) is located more northward, and at the south of the region of maximum convergence, enhanced rainfall is evident^15^. Also, from Fig. 1B (left panel), at about between 0° to 10°N, another region of maximum convergence extends from the tropical Atlantic Ocean to the Southern parts of Nigeria. Hence, given that the northeasterly wind that penetrates the northern parts of Nigeria is dry, in the climatology, from Fig. 1B (right panel) the mean rainfall amount in Nigeria is higher in the southern parts relative to the northern parts.

Studies have addressed rainfall variability and change in Nigeria. Among such studies^14^, investigated the variability of West African summer monsoon rainfall. Adedoyin^17^ analyzed the relationship between global SSTa and rainfall in northern Nigeria. Tarhule and Woo^18^ analyzed trends in rainfall characteristics in northern Nigeria. Adelekan^19^ found that up to six regions in Nigeria exhibit internal coherency in terms of the fluctuations of thunderstorm rainfall over time. Nnamchi et al.^20^ investigated the spatial patterns of the twentieth century mean seasonal precipitation over West Africa.

Limited studies have addressed the climatic processes driving rainfall changes in Nigeria. Among such studies^21^, linked the leading patterns of summer precipitation over West Africa to global teleconnections. Nnamchi and Li^22^ investigated the link between the South Atlantic Ocean dipole and the interannual rainfall variability over West Africa during the boreal summer monsoon rainy season. Bliefernicht et al.^23^ investigated the circulation patterns linked to heavy rainfall in West Africa. Bárdossy^24^ explored the association between the West African monsoon's onset and atmospheric circulation patterns. Given the spatial heterogeneity of rainfall, there is the need to (1) understand the rainfall coherent regions in Nigeria as well as the atmospheric circulation patterns associated with wet and dry regimes over the rainfall coherent regions; and (2) investigate the linkages between changes in the amplitude of the atmospheric circulation patterns associated with the development of the rainfall coherent regions and long-term rainfall changes over the associating rainfall regions in Nigeria. To address these two concerns, we regionalize rainfall in Nigeria during the rainy season and analyze the atmospheric circulation patterns that drive dry and wet regimes over the rainfall coherent regions in Nigeria. Further, we examined trends in rainfall as well as the atmospheric circulation patterns contributing to the trends. Therefore, our research goals are to improve the understanding of the large-scale circulation patterns driving spatiotemporal rainfall variability patterns in Nigeria during the rainy season as well as link changes in the amplitude of the circulation patterns to historical trend in rainfall over specific regions in Nigeria.

## Results and discussion

### Rainfall variability patterns in Nigeria during the rainy season

**Figure 2:**
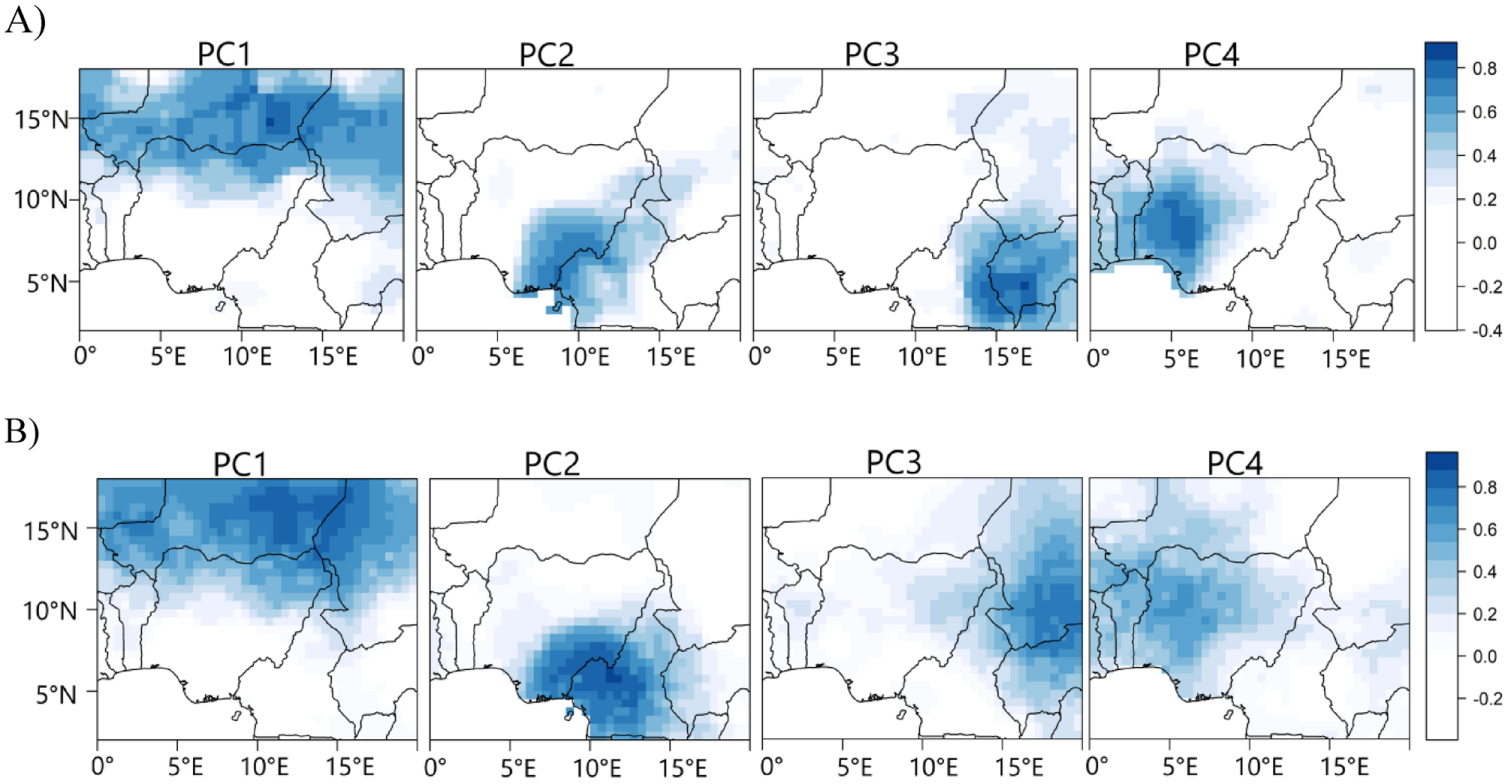
Rainfall variability patterns from CPC (**A**) and the UDEL (**B**) rainfall products during July to September. Color is the rotated PC loadings. The rainfall variability patterns are based on Promax rotated principal component analysis. Figure 2 was created using R studio version 4.2.0 available at https://cran.microsoft.com/snapshot/2022-04-25/bin/windows/base/.

Figure 2 shows the rainfall variability patterns associated with the four retained and rotated principal components (PCs), both from the CPC and UDEL data. PC1, which explains 24% of the variance, regionalizes the northern parts of Nigeria; PC2, which explains 17% of the variance, regionalizes the southeastern parts of Nigeria and PC4, which explains 8% of the variance, regionalizes the western parts of Nigeria. Given the impreciseness of the climatic processes associated with rainfall variability in the classified rainfall coherent regions in Fig. 2, the spatially simplified PC loading patterns suggest that the central parts of Nigeria cannot be explicitly classified. This implies that rainfall over the central parts of Nigeria can exhibit similar rainfall characteristics of the northern, western, and southeastern rainfall coherent regions. Moreover, based on the differing loading magnitudes under each variability pattern, the extent to which rainfall anomaly is coherent under a given homogeneous region is different, which can be linked to differences in localized factors that contribute to rainfall formation. PC3, which explains 13% of the variance, mainly regionalizes rainfall in the neighboring countries east of Nigeria.

The representation of rainfall over the tropics is challenging^25^, even among gridded observational data^26^. Hence to ensure the robustness and validity of the classified variability patterns, we have applied the classification to several gridded rainfall products, such as those available at https://psl.noaa.gov/data/gridded/tables/precipitation.html. The variability patterns in Fig. 2 were reproduced by the gridded datasets but with inconsistencies in the spatial structure of the patterns. The most consistent variability patterns from the distinct rainfall products were obtained from CPC and UDEL rainfall products, though UDEL exists at a shorter time frame compared to CPC, which is up to date. Nonetheless, Fig. 2 shows that there is a good resemblance between the variability patterns from CPC and UDEL, with a congruence match greater than 0.93 for all PCs except for PC3 which has a congruence match of 0.89. However, within the borders of Nigeria, the two rainfall products agree that July to September (JAS) rainfall can be regionalized into the northern, western, and southeastern regions, with an overlap in the central parts of Nigeria (Fig. 2). Moreover^27^, reported that the CPC rainfall data performs relatively well in reproducing rain-gauged data over parts of the African domain. Given that the CPC rainfall data is available for a longer period, subsequent reference and analysis of the rainfall variability patterns in Fig. 2 will be for patterns derived from the CPC data.

**Figure 3:**
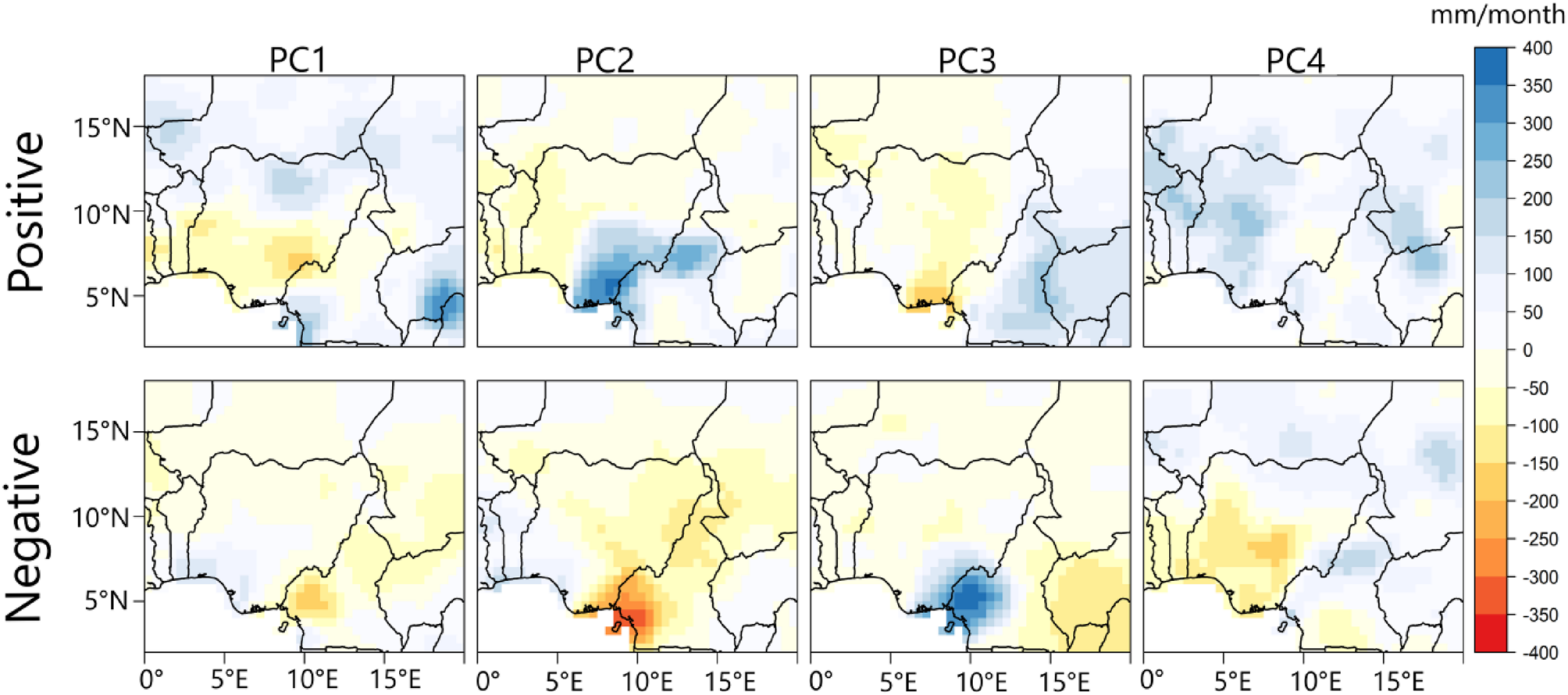
Composite anomaly patterns of rainfall associated with the positive and negative phases of the rainfall variability pattern in Fig. 2. The anomaly patterns were calculated as the difference between the clustered dates when the rainfall regimes were most expressed and the JAS climatology. The clustered dates are presented in Table A1. Figure 3 was created using R studio version 4.2.0 available at https://cran.microsoft.com/snapshot/2022-04-25/bin/windows/base/.

The PC scores comprise both negative and positive values which can be advantageous in recovering the asymmetry in the rainfall variability patterns in Fig. 2. Hence to uncover which phase of the PC scores is associated with the wet or dry regime of the patterns in Fig. 2, we examined the composite rainfall anomaly patterns using a cluster of dates when the positive and negative phases of the PC scores have values that are large enough (generally 1.2 in this study) to reproduce the corresponding pattern configuration in Fig. 2. Figure 3 shows the results—we have structured the positive phase of the scores to represent the wet regimes and the negative phase to represent the dry regimes—by multiplying the PC score values by minus 1 when the negative phase contains rather the wet regime. From Fig. 3, in the positive (negative) phase of the four PCs, PC1 indicates positive (negative) rainfall anomaly in the northern parts of Nigeria; PC2 indicates positive (negative) rainfall anomaly in the southeastern parts of Nigeria, and PC4 indicates positive (negative) rainfall anomaly at the western parts of Nigeria. Similarly, PC3 indicates positive (negative) rainfall anomaly in the neighboring countries, east of Nigeria.

**Figure 4:**
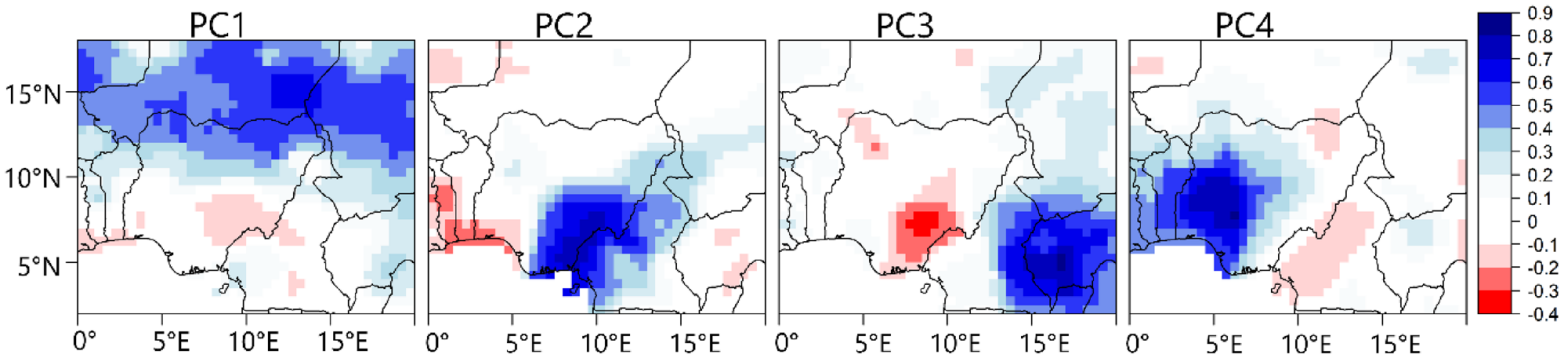
Correlation map between the temporal variability patterns of the PCs (i.e., the PC scores) and rainfall anomaly field from 1979 to 2022 during JAS. Color is the Pearson correlation coefficient. Figure 4 was created using R studio version 4.2.0 available at https://cran.microsoft.com/snapshot/2022-04-25/bin/windows/base/.

To further examine the validity of the patterns in Fig. 3 as well as how the re-structured PC scores recover the asymmetry in the variability patterns in Fig. 2—i.e., correctly detecting the wet and dry regimes under a given rainfall coherent region—we applied correlation maps as shown in Fig. 4. We expect positive correlation coefficient values over the coherent regions in Fig. 2, supporting that the positive phase of the PC scores is associated with rainfall formation in the rainfall coherent region under investigation. From Fig. 4, under PC1, a positive correlation between the PC scores of PC1 and the rainfall anomaly field is evident in the northern parts of Nigeria and a similar argument holds for the other PCs supporting the validity of the (re-structured) PC scores in recovering the wet and dry regimes under a given rainfall coherent region in Fig. 2.

**Figure 5:**
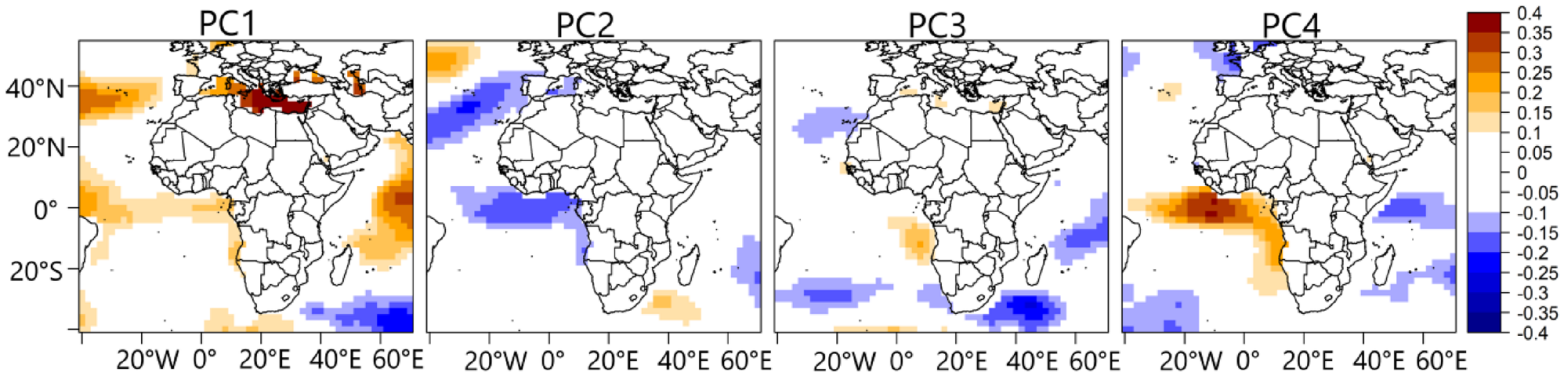
Correlation map between the temporal variability patterns of the PCs (i.e., the PC scores) and sea surface temperature anomaly from 1979 to 2022 during JAS. Color is the Pearson correlation coefficient. Figure 5 was created using R studio version 4.2.0 available at https://cran.microsoft.com/snapshot/2022-04-25/bin/windows/base/.

Further, Figs. 5 and A1 show the correlation between the (re-structured) PC scores and SSTa. Similar to Fig. 4, we interpret positive correlation to imply that above-average (below-average) SSTa is associated with rainfall increase (decrease) over the rainfall coherent region in question; and negative correlation to imply that above-average (below-average) SSTa is associated with rainfall decrease (increase) over the rainfall coherent region in question.

**Figure 6:**
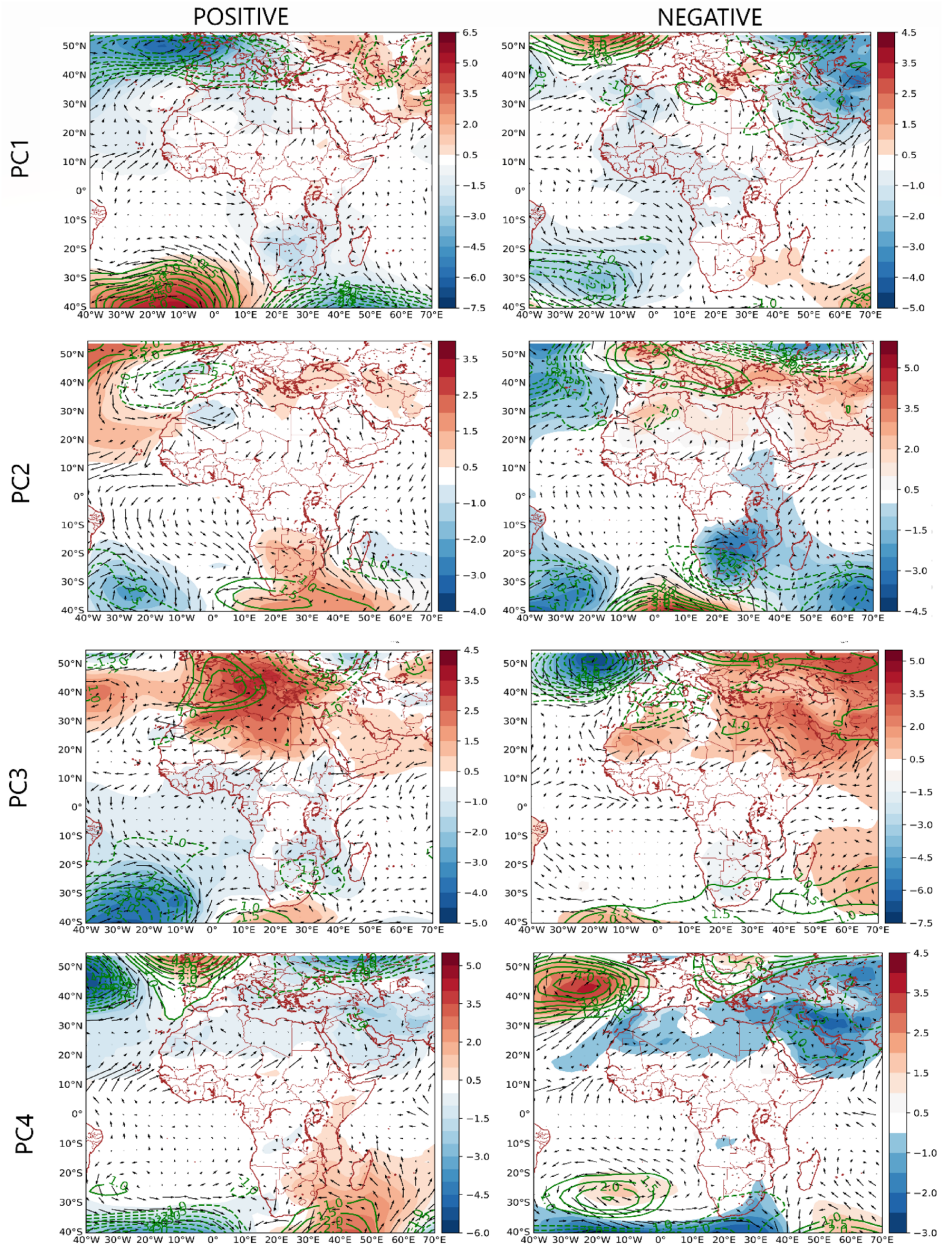
Composite anomaly maps of sea level pressure (color), 700 hPa height, and 850 hPa moisture flux (black vectors) associated with the variability patterns in Fig. 2. Only statistically significant values based on the permutation test were plotted. Anomalies are calculated with respect to the JAS climatology. Contour interval is 0.5 m. Thick (dashed) contours represent positive (negative) height values at 700 hPa. Figure 6 was created using python 3.8.8 available at https://www.python.org/downloads/release/python-388/.

**Figure 7:**
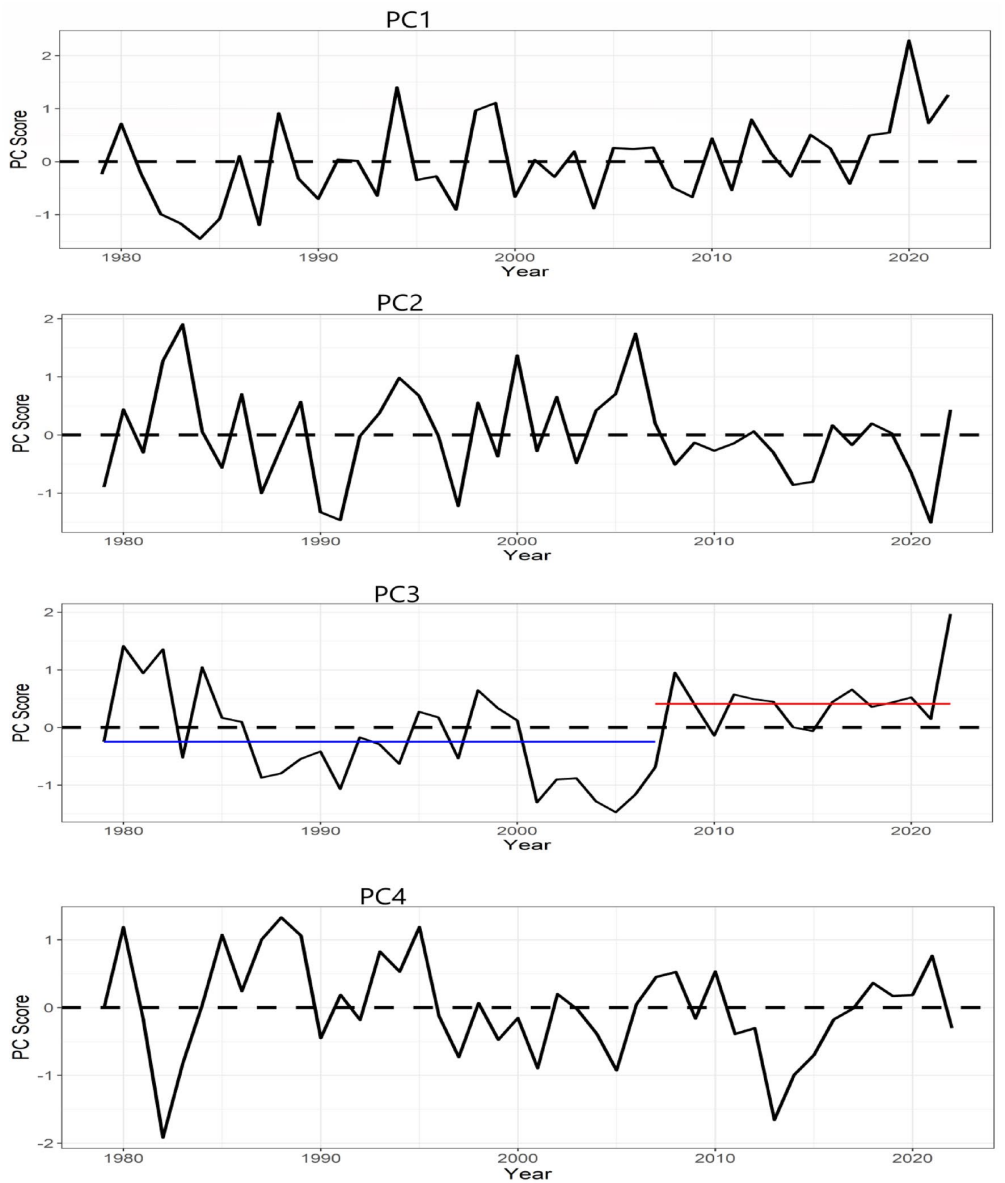
Inter-annual averages of the amplitude of the rainfall variability patterns in Fig. 2. The blue and red horizontal lines indicate that after 2007, there is an abrupt shift in the mean amplitude of PC3, based on Pettitt’s test for single change-point detection, Fig. 7 was created using R studio version 4.2.0 available at https://cran.microsoft.com/snapshot/2022-04-25/bin/windows/base/.

Table 1 contains the correlation between the PC scores and teleconnection patterns (i.e., climate indices) that modulate the regional circulation patterns associated with the time development of the rainfall regimes. Figures 6 and 7 show the composite anomaly patterns of SLP, 700 hPa height and 850 hPa moisture fluxes (used to characterize low level convergence), and the inter-annual mean amplitude of the patterns in Fig. 2, respectively. Under PC1 Figs. 5 and A1 indicate that SSTa over the global oceans (i.e., the Indian, Pacific, and Atlantic) oceans and the Mediterranean Sea are associated with rainfall variability over the northern region (i.e., PC1). Specifically, positive SSTa over the Mediterranean Sea, parts of the North Atlantic and South Atlantic Ocean, the tropical Indian Ocean and the western tropical Pacific correspond with rainfall increase over the northern region (PC1). Similarly, negative SSTa at the southwest Indian Ocean, the eastern part of the tropical Pacific Ocean, and over the Southern Ocean (south of Australia) correspond with rainfall increase in the northern region (PC1). The zonal dipole pattern over the tropical north Pacific whereby the western part of the Pacific is warmer, and the eastern part is colder (Figure A1, PC1) suggests an association between the negative phase of the Pacific Decadal Oscillation (PDO) and rainfall formation over the northern region (PC1). The results in Table 1 confirm also that PC1 is negatively correlated with the PDO, validating that the negative phase of the PDO is associated with rainfall increase over the coherent regions in northern Nigeria. PC1 is equally associated with other SST modes over the global oceans, supporting the results in Figure A1. Specifically, negative (positive) phases of the North Atlantic Oscillation, and the North Pacific Oscillation are associated with rainfall increase (decrease) over the northern part of Nigeria (Table 1). Similarly, the positive (negative) phases of the Atlantic Multidecadal Oscillation and the Pacific warm pool are associated with rainfall increase (decrease) over the northern part of Nigeria. PC1 also has the strongest relationship with rainfall variations over the Sahel and the Pacific warm pool. Ref^17^ also reported the influence of SSTa in the global oceans on rainfall in northern Nigeria.

**Table 1:** Correlations between the temporal variability patterns of the PCs in Fig. 2 (i.e., the PC scores) and climate indices.

Climate index	PC1	PC2	PC3	PC4
North Atlantic Oscillation	$-$ 0.23			
Atlantic Multidecadal Oscillation	0.28			
North PACIFIC oscillation	$-$ 0.21			
Tropical Southern Atlantic				0.23
Pacific Decadal Oscillation	$-$ 0.29			
Pacific Warm Pool	0.34			
ENSO precipitation index			$-$ 0.22	
Sahel rainfall	0.55			
Southern Oscillation			0.27	
Global Mean Land/Ocean Temperature	0.31			

Only statistically significant correlations at a 95% confidence level based on the Kendall Tau-b are reported.

From Fig. 6, rainfall formation in the northern parts of Nigeria (i.e., the positive phase of PC1) is associated with the dominance of low-level cyclonic circulation, up to 700 hPa, over the North Atlantic Ocean and parts of the Mediterranean, coupled with a weaker low situated over parts of the Western Asian landmass; this circulation pattern weakens the intrusion of dry northerly air from the Sahara into the northern parts of Nigeria^28,29^. From the negative phase of PC1, dry conditions in the northern parts of Nigeria are linked to the dominance of low-level cyclonic circulation, up to 700 hPa, over parts of the Western Asian landmass favoring the penetration of northeasterly winds from the Sahara into the northern parts of Nigeria^28,29^. From Figure A2, moisture flux over Nigeria has northerly (southerly) component during the dry (wet) regime of the northern region (PC1). Also, from the JAS SLP and rainfall climatology in Fig. 1B, the cyclonic anomaly situated over Western Asia contributes to the circulation anomaly resulting in why in the rainfall climatology, the northern parts of Nigeria are relatively drier than the southern parts. Moreover, from Fig. 3, under PC1, there is a meridional dipole rainfall pattern such that when the northern part of Nigeria is wet, the southern parts become dry and vice versa. Figure 6 (PC1 positive phase and PC3 negative phase) indicates that this is because cyclonic circulation over the North Atlantic and parts of the Mediterranean might co-occur with the enhancement of the low over Angola and northern Mozambique, as well as the enhancement of low-level anticyclonic circulation (up to 700 hPa) over the subtropical south Atlantic high-pressure, thus, disrupting the penetration of moist westerly and southerly winds into southern Nigeria. This suggests that extra-tropical forcing can influence tropical rainfall regions.

From Table 1 no significant correlation was found between the southeastern region (PC2) and the climate indices. From Fig. 5, cold SSTa over the tropical Atlantic Ocean, south coast of Nigeria (i.e., the Gulf of Guinea), is associated with rainfall formation in the southeastern parts of Nigeria (PC2). Figures 6 and A2 shows that anticyclonic circulation anomaly at the western branch of the Mascarene high as well as in the tropical Atlantic Ocean, south coast of Nigeria, favors the penetration of southwesterly moisture fluxes into the southeastern parts of Nigeria bringing about rainfall formation. Conversely, in the absent of anticyclonic circulation over the tropical Atlantic Ocean, south coast of Nigeria, the penetration of moist winds from the tropical Atlantic Ocean into the southeastern parts of Nigeria is weakened (Fig. A2, PC2). Moreover, under the negative phase of PC2, there is enhanced intrusion of dry northerly air into large parts of Nigeria, including the southeastern parts, suppressing moist convection (Figs. 6 and A2). Unlike the southeastern part of Nigeria (PC2), for the western parts of Nigeria (PC4), Figs. 3 and 5 show that warm SSTa at the tropical Atlantic Ocean, south coast of Nigeria (i.e., the Gulf of Guinea), rather brings rainfall formation over the western parts of Nigeria (PC4). This is because warm SSTa at the tropical Atlantic Ocean, south coast of Nigeria, implies that southerly wind advects more moisture from the South/tropical Atlantic Ocean into the western parts of Nigeria (Figs. 6 and A2, PC4). However, colder SSTa at the tropical South Atlantic Ocean corresponds with southerly winds transporting relatively drier moisture into the western parts of Nigeria (Fig. A2, PC4). From Table 1, rainfall variability in the western parts of Nigeria is positively related to the Tropical Southern Atlantic index. Specifically, the positive (negative) phase of the Tropical Southern Atlantic index is associated with rainfall (increase) in the western part of Nigeria.

### Linear trends in the circulation patterns associated with the rainfall regimes

**Figure 8:**
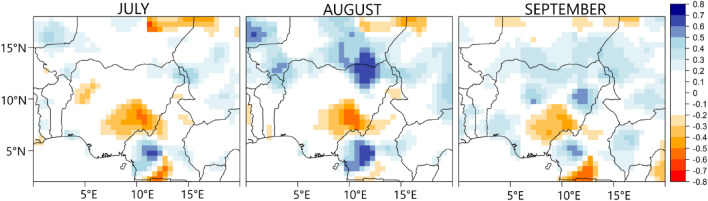
Linear trends in rainfall during July, August, and September from 1979 to 2022. Color is the standardized regression coefficient. Figure 8 was created using R studio version 4.2.0 available at https://cran.microsoft.com/snapshot/2022-04-25/bin/windows/base/.

**Figure 9:**
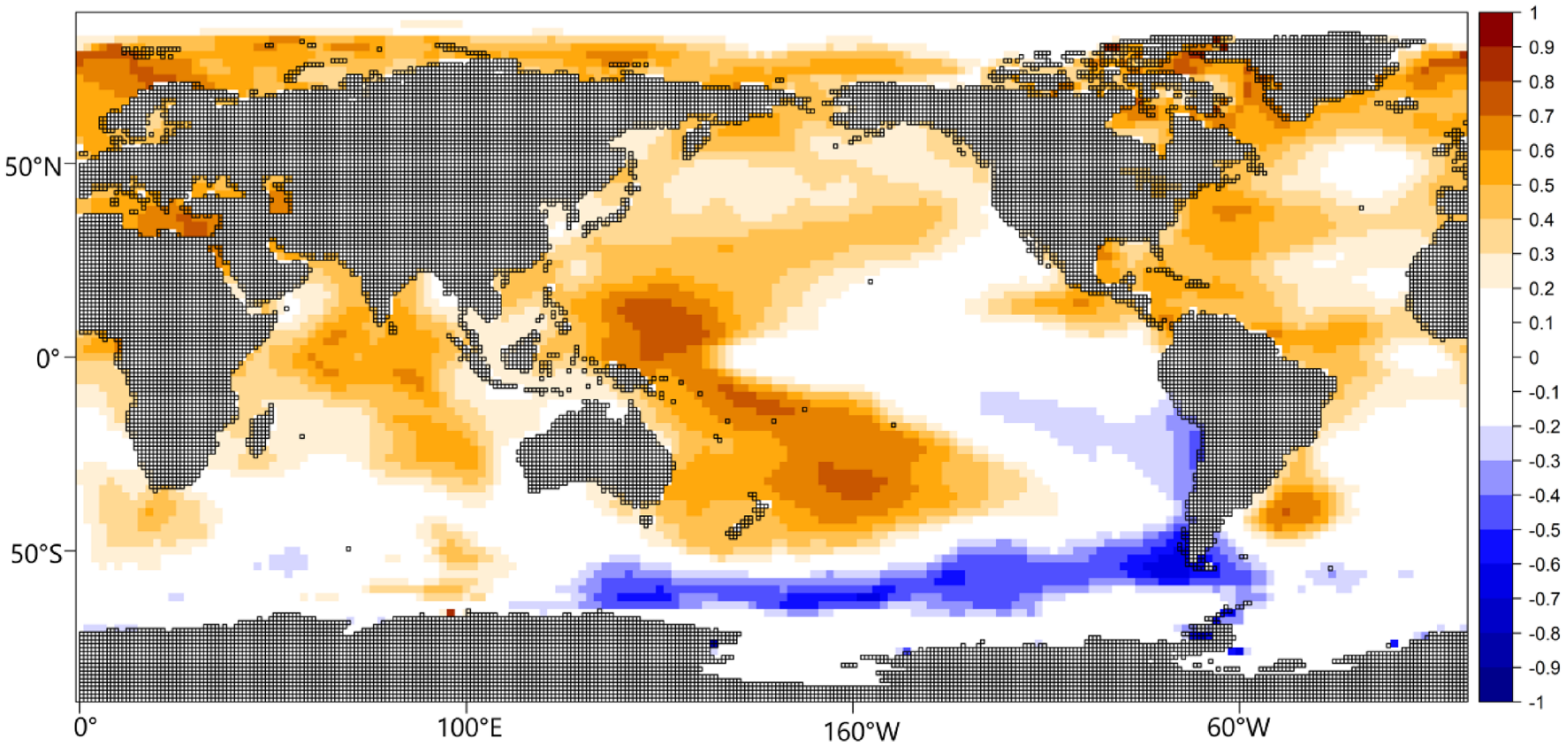
Linear trends in sea surface temperature anomaly during JAS from 1979 to 2022. Color is the standardized regression coefficient. Figure 9 was created using R studio version 4.2.0 available at https://cran.microsoft.com/snapshot/2022-04-25/bin/windows/base/.

From Fig. 7, the amplitude of PC1 (the northern region) shows a positive significant trend based on the modified Mann–Kendall test for trends^30^, i.e. a temporal shift in the asymmetric circulation pattern towards the wet regime. Consequently, from Fig. 8, the rainfall trend is positive in the northern part of Nigeria during JAS. Since during the wet regime of the northern region (PC1) SSTa is warm over the North Atlantic Ocean, the Mediterranean Ocean, and the western Pacific Ocean (Figure A1), the positive trend in SSTa over the aforementioned oceanic regions (Fig. 9) possibly contributes to the strengthening of the wet regime of the northern region (PC1) and its circulation features (in Fig. 6), thereby resulting to a positive trend in rainfall at the northern parts of Nigeria (Fig. 8). Based on regression analysis and the CPC data, rainfall significantly increases over the northern parts of Nigeria, during the rainy season, by 2 to 4 mm/year—with the highest increase during August. Moreover^31^, reported that rainfall is increasing over the Sahel owing to the northward shift of the ITCZ over West Africa. Rainfall in the northern parts of Nigeria is strongly coupled with rainfall at the Sahel (Table 1), therefore it can be inferred that increasing SSTa at the tropical/subtropical oceans of the Northern hemisphere (Fig. 9) contributes to the northward shift in the mean position of the ITCZ and the region of maximum rainfall in Fig. 1, resulting to increasing rainfall in the northern region. Huang et al.^32^ reported that the position of the ITCZ can be modulated by regional SSTa. Because the ocean and the atmosphere are slightly warmer in the Northern hemisphere, over the tropics, the mean position of the latitude of maximum precipitation is located more north of the equator ^33,34^. Similarly^35^, showed that cooling (warming) of the Northern hemisphere (southern hemisphere) caused the ITCZ to displace southward and vice versa. Therefore, as the tropical/subtropical oceans of the Northern Hemisphere warm, the northern parts of Nigeria are likely to become wetter.

At a 95% confidence level, we did not find any statistically significant trend in the temporal patterns of the other PCs, however, under PC2 (i.e., the southeastern region), there is an indication of a temporal shift towards the dry regime, during the end of the analysis period (Fig. 7). Consequently, the rainfall trend in the southeastern part of Nigeria is negative (Fig. 8), and with a significant decrease of about 5 mm/year based on the linear regression analysis. Since from Fig. 5, colder SSTa at the tropical South Atlantic Ocean is required for rainfall formation in the southeastern region (PC2), from Fig. 9, the positive trend in SSTa at the tropical South Atlantic Ocean (i.e., over the Gulf of Guinea) possibly contributes to the strengthening of the dry regime of the southeastern region and its circulation features, thereby resulting to negative trend in rainfall at the southeastern region of Nigeria (Fig. 8).

## Conclusions

We analyzed the rainfall variability patterns during the rainy season in Nigeria. Three rainfall coherent regions were classified within Nigeria during JAS. The rainfall coherent regions comprised the northern, western, and southeastern parts of Nigeria. Rainfall in the northern part of Nigeria, during the rainy season, was found to be influenced by SSTa in the global oceans, while rainfall in the western and southeastern parts of Nigeria is mostly associated with SSTa in the tropical Atlantic Ocean, south coast of Nigeria. Also, the hydroclimate of northern parts of Nigeria was found to be strongly coupled with rainfall variations at the Sahel. Positive SSTa over the Mediterranean and the adjacent oceans, coupled with the weakening of northerly winds from the Sahara penetrating northern Nigeria, were found to be major mechanisms associated with rainfall formation over the northern parts of Nigeria. Positive SSTa at the tropical Atlantic Ocean, south coast of Nigeria, was found to be a major mechanism associated with rainfall formation over the western parts of Nigeria. For the southeastern parts of Nigeria, cold SSTa over the tropical Atlantic Ocean, south coast of Nigeria, is associated with rainfall formation in the southeastern parts of Nigeria. Further, during the analysis period, we found a positive significant trend in JAS rainfall in the northern parts of Nigeria and a negative significant trend in JAS rainfall in the southeastern parts of Nigeria, which were linked to changes in global SSTa.

## Data and methodology

### Data

The climate data sets used in this work are reanalysis products from NCEP-NCAR^36^ and ERA5^37^; and observed gridded rainfall products from the Climate prediction center (CPC)^38^ and UDEL^39^. The reanalysis data sets are rainfall, sea level pressure (SLP), 10-m wind vectors, 850 hPa wind vectors, specific humidity, 700 hPa height, and vertical velocity. The reanalysis data sets were obtained at a horizontal resolution of 2.5° $\times$ 2.5°, and 0.25° $\times$ 0.25° longitude and latitude for NCEP and ERA5, respectively; and from 1979 to September 2022. The temporal resolution of the data sets is monthly. The composite patterns from NCEP were used to externally validate the same composite patterns from ERA5. Rainfall data from CPC spans from 1979 to September 2022 and from 1979 to 2014 for the UDEL rainfall data. Both CPC and UDEL rainfall data sets have a horizontal resolution of 0.5° longitude and latitude. SST data is obtained from NOAA Extended Reconstructed SST version 5^40^ for 1979 to 2022.

### Ethics approval

No human subject is involved in this study and Figures belong to the author. The paper is also not under consideration in any Journal. There is also no conflict of interest in this paper.

### Consent to participate

No human research is used. The author consent this paper to be considered.

### Classification of rainfall regimes

For the classification of the rainfall coherent regions in Nigeria during the rainy season, we applied the fuzzy rotated S-mode (i.e., the column contains grid points, and the row contains times) principal component analysis^41,42^. Nigeria averages the highest rainfall amount from July to September; therefore, to obtain more robust signals associated with rainfall variability during the rainy season in Nigeria, we applied our climate classification algorithm to observed rainfall data from CPC and UDEL for July to September months. Before the classification, the annual cycle of the rainfall data was removed. Since the physical processes associated with the development of climatic regions are continuous and do not have step boundaries, we have included the neighboring countries in Nigeria, when selecting the spatial domain for classifying rainfall coherent regions in Nigeria.

The correlation matrix is used to obtain the relationship between observed rainfall fields at the grid points. Afterward, the correlation matrix is decomposed using the singular value decomposition resulting in the principal component scores, eigenvectors, and eigenvalues. The eigenvectors are spatial variability patterns, and the principal component scores designate the amplitude of the patterns at a given time. To make the eigenvectors become correlations between the PC scores and the standardized rainfall anomaly field, the eigenvectors were multiplied by the square root of the corresponding eigenvalues, resulting in principal component loadings that are more responsive to simple structure rotation^41,43^ and can be longer than a unit of length. In deciding the number of PCs to retain and rotate, we consider that the rotation aims to enhance the physical interpretability of the PCs, which is measured by the degree to which all the rotated principal components resemble the patterns embedded in the correlation matrix from where they are calculated^43^. Thus, by retaining iteratively 2 principal components and above, we assess the number of rotated principal component at which every principal component loading, matches the vector in the correlation matrix that has the highest principal component loading magnitude, for that principal component, with a congruence (coefficient) match of at least 0.92 (i.e., a good match). For that purpose, we use the Promax rotation^44^ at a power of 2 (i.e., the power at which the Varimax solution is raised). The Promax rotation is an oblique rotation that relaxes orthogonality at the principal component scores while maximizing the number of near-zero loadings and fewer larger loadings, resulting in unique rainfall patterns of variability. In this study, following the criterion of interpreting only principal components that have a good congruence match with the patterns embedded in the correlation matrix, four principal components were retained in the region of assessment.

After deciding the number of principal components to rotate that best represents the signals embedded in the correlation matrix, those principal components are deemed physically interpretable and were analyzed further to uncover the circulation patterns associated with the time development of the regions as well as the association between the rainfall variability patterns and global SSTa modes of variability and all the teleconnection patterns available at https://psl.noaa.gov/data/climateindices/list/ from 1979 to 2022, during JAS. The association between the classified rainfall variability patterns and climate indices was examined using correlation analysis—i.e., by correlating the principal component scores and climate index. The correlations were tested for statistical significance at a 95% confidence level. The principal component scores are used in grouping dates to create composite anomaly maps of SLP, 700 hPa height, and moisture flux for each rainfall variability pattern. This was achieved by examining the PC score magnitude—for the wet and dry phase of the rainfall pattern, respectively—with clustered dates resulting in composite anomaly rainfall maps that match with the principal components loadings (i.e., the associating spatial variability patterns) with a congruence match of at least 0.92. Table A1 contains the clustered dates used in creating the composite anomaly maps under each PC.

We examined rainfall trends at each grid point and the associating variability pattern associated with the trend (i.e., trends in the PC scores). Following the classification of the rainfall variability patterns and applying the principal components score in uncovering the circulation patterns associated with each classified rainfall coherent region, in the process, we also determined the asymmetric patterns associated with wet and dry regimes in each rainfall coherent region.

## Supplementary Information

Supplementary Information.

## Data Availability

The CPC, UDEL, NCEP, and NOAA SST data sets are obtained from https://psl.noaa.gov/data/gridded/. NCEP-NCAR and ERA5 reanalysis data sets are available at https://psl.noaa.gov/data/gridded/data.ncep.reanalysis.html and https://climate.copernicus.eu/climate-reanalysis, respectively.

## Code availability

R studio was used for coding the methods as described in the methodology section. The codes are base packages in R (e.g. the PROMAX routine).
