# Supplementary material for: Rainfall variability patterns in Nigeria during the rainy season
Source: Sci Rep. 2023 May 16;13:7888. doi: 10.1038/s41598-023-34970-7 (PMC10188553; doi:10.1038/s41598-023-34970-7)
Supplement: Supplementary file 1 — Supplementary Information. [file 41598_2023_34970_MOESM1_ESM.docx]

Appendix


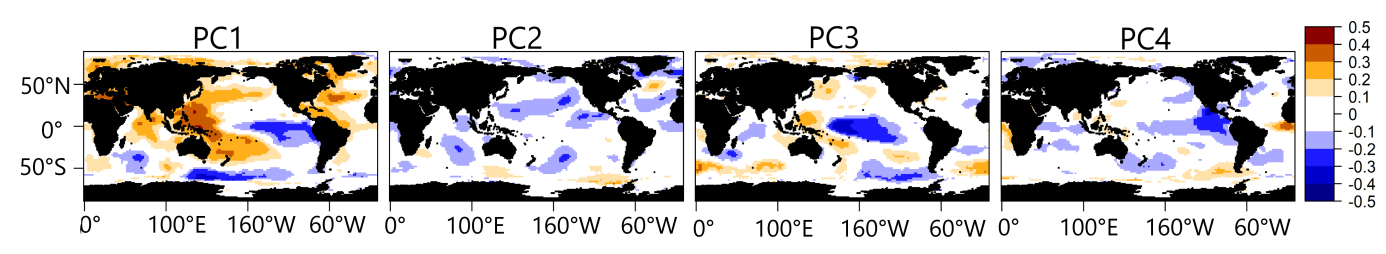


Figure A1: correlation between sea surface temperature anomalies and the PC scores of the rainfall variability patterns in Figure 2


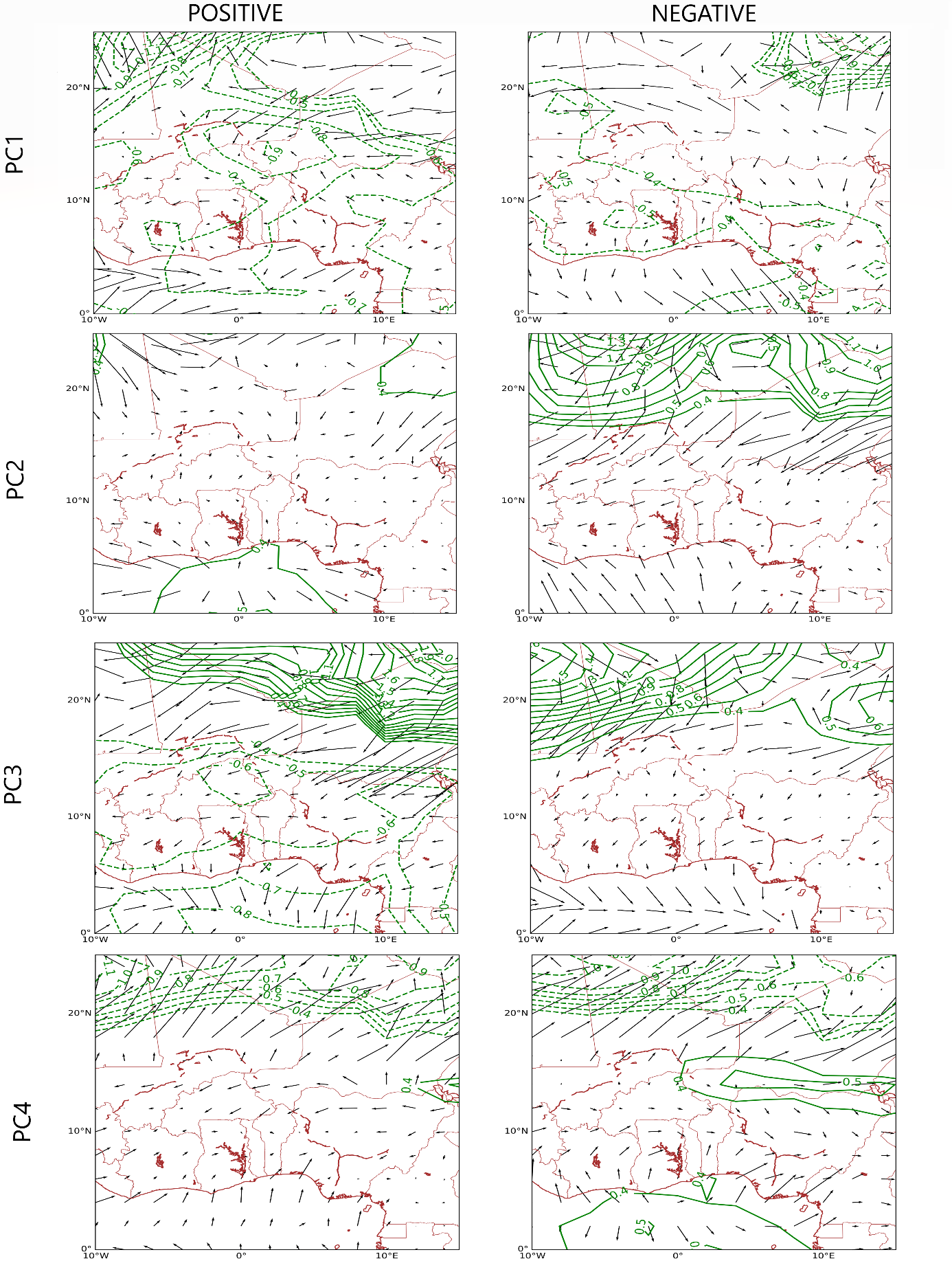


Figure A2: Composite anomaly patterns of SLP (green contour lines) and 850 hPa moisture flux (black vectors) for the rainfall variability patterns in Figure 2. The maps are the same as in Figure 6 but with the spatial domain more focused towards Nigeria. Contour interval is 0.1 hPa

Table A1: Dates clustered under the phase of a given PC. The dates were used to create the composite maps in Figures 3 and 6

| PC1+ | PC1- | PC2+ | PC2- | PC3+ | PC3- | PC4+ | PC4- |
| --- | --- | --- | --- | --- | --- | --- | --- |
| 1994-08 | 1982-07 | 1982-09 | 1981-08 | 1980-09 | 1990-07 | 1980-08 | 1982-07 |
| 1998-09 | 1983-07 | 1983-08 | 1987-09 | 1984-07 | 2001-08 | 1988-08 | 1982-08 |
| 2019-08 | 1983-08 | 2000-07 | 1990-08 | 2022-07 | 2002-09 | 1991-07 | 1982-09 |
| 2020-08 | 1984-08 | 2005-09 | 2020-08 | 2022-09 | 2003-08 | 1995-08 | 2013-08 |
| 2021-07 | 1985-08 | 2006-08 |  |  | 2004-08 | 2021-08 | 2014-07 |
|  | 1987-08 | 2006-09 |  |  | 2005-09 |  | 2020-08 |
|  |  |  |  |  | 2006-09 |  |  |
